# Screening of proteases produced by *Aspergillus* micromycetes active against proteins of the hemostasis system

**DOI:** 10.18502/CMM.2023.150674

**Published:** 2023-03

**Authors:** Daria Surkova, Viktoria Lavrenova, Sergey Klyagin, Anna Shestakova, Alexander Osmolovsky

**Affiliations:** 1 Department of Microbiology, Faculty of Biology, Lomonosov Moscow State University, Moscow, Russia; 2 Faculty of Biology and Biotechnology, HSE University, Moscow, Russia

**Keywords:** *Aspergillus*, Biotechnology, Fibrinolytic enzymes, Fungal proteases, Hemostasis

## Abstract

**Background and Purpose:**

For these intents, proteases cardiovascular disease is the primary cause of death; hence, accurate diagnosis and treatment are urgently required are regarded as prospective agents . High substrate specificity is needed for an effective enzyme, which makes *Aspergillus* micromycetes, known for producing proteases with precise action, biotechnologically promising. This study mainly aimed to look at the possibilities of *Aspergillus* species, which had never been mentioned in terms of general proteolytics.

**Materials and Methods:**

Every species was cultivated in two-stage submerged conditions with two different nitrogen sources; whereupon, proteolytic activity in culture fluid was determined. Chromogenic peptide substrates and fibrin plates were used to evaluate the thrombin, plasmin, factor Xa, urokinase, protein C-like, activating activities towards hemostasis proteins, as well as fibrinolytic and plasminogen-activating activities of these species.

**Results:**

It was found that *A. aureolatus* and *A. tennesseensis* are active proteolytics exhibiting plasmin-like activities (116.17 and 87.09 U×10^-3^, respectively), factor Xa-like activity (76.27 and 77.92 U×10^-3^, respectively) and urokinase activity (85.99 and 59.91 U×10^-3^, respectively). The thrombin-like activity was found for *A. tabacinus* (50.37 U×10^-3^), and protein C-like activity was noticeable for *A. creber*, *A. jensenii*, *A. protuberus*, and *A. ruber* (62.90, 65.51, 73.37, and 111.85 U×10^-3^, respectively). Additionally, more than half of species had the ability to directly activate plasminogen or operate as fibrinolytics.

**Conclusion:**

New proteolytic strains were discovered, offering hope for the therapy of cardiovascular disorders. The high specificity and activity of fungal enzymes make them useful in a variety of fields, including medicine and diagnostics.

## Introduction

Year after year, cardiovascular diseases (CVDs) are reported by WHO as a main death cause worldwide. Stroke and heart attacks account for 85% of all CVD deaths [ 1 ]. One of the most common conditions is associated with hemostasis disorders, such as intravascular thrombosis and thromboembolism. Therefore, their diagnosis and treatment are urgent and should be accessible to all those in need. The CVD enzyme therapy is considered a prominent approach, as the hemostasis system represents several proteolytic reactions, connected and dependent on each other [ 2 ].

Although there are new artificial oral anticoagulants and recombinant human proteins (tenecteplase and alteplase), the investigation of microbial proteases active against components of the human hemostasis system attracts considerable attention [ 3 ]. They are regarded due to high activity, selectivity, and stability, as well as reduced price (compared to complicated novel drugs), and hereby, they are available, especially for patients from developing countries with low spending on healthcare systems and medicine. 

Many microbial proteases which are active against human hemostasis proteins are well known and have been investigated. Their activity may be expressed as direct (when the enzyme disrupts the thrombi itself) or activating (when it controls coagulation by activating and deactivating proteolytic cascades through limited proteolysis) [ 4 ]. Both bacteria and microscopic fungi can produce and secrete demanded enzymes. The former usually secrete a few (1-3) proteases, while the latter secretes more (up to 12) [ 5 , 6 ]. This enables micromycetes to narrow the substrate specificity of secreted enzymes, which is a crucial characteristic for the medical application of microbial proteases. 

One of the genera of prospective producers is *Aspergillus*, which is known as an effective protease producer, in particular with activities similar to those involved in hemostasis [ 3 , 7 - 9 ]. Some investigated species show direct fibrinolytic (*A. niger* and *A. flavus*), plasmin-like (*A. flavipes* and *A. sydowii*), plasminogen-activating (*A. nidulans*), factor X-activating (*A. ochraceus*), and other activities [ 10 - 13 ]. However, there is a constant search for new producers and enzymes, as less toxic and allergenic (and nevertheless more active and specific) enzymes can be found. 

In this work, a screening on several activities (thrombin-, plasmin-, factor Ха-, urokinase-, and protein C-like) was performed for 15 *Aspergillus* species, whose proteases were not previously described.

## Materials and Methods

### Chemicals and reagents

All inorganic compounds and starch were purchased from Chimmed Company, Russia. Moreover, peptone and fish flour hydrolysate were obtained from Helicon Company, Russia. All chromogenic substrates were purchased from Chromogenix Company, Spain. In addition, bovine fibrinogen was purchased from Merck Company, USA, and human blood plasma was purchased from Renam company, Russia.

### Fungal species

In total, 15 different species of *Aspergillus* genus from the Mycotheca of the Faculty of Biology (BEOFB), University of Belgrade, Belgrade, Serbia, were used for proteolytic potential assessment. They included *A. aureolatus* BEOFB3320m, BEOFB *A. calidoustus* BEOFB3220m, *A. creber* BEOFB3250m, *A. europaeus* BEOFB382m, *A. jensenii* BEOFB3200m, *A. melleus* BEOFB3180m, *A. penicilloides* BEOFB3190m, *A. proliferans* BEOFB3280m, *A. protuberus* BEOFB3240m, *A. pseudoglaucus* BEOFB3170m, *A. domesticus* BEOFB3270m, *A. ruber* BEOFB3150m, *A. tabacinus* BEOFB3260m, *A. tennesseensis* BEOFB3310m, *A. tubingensis* BEOFB3300m. These strains were previously isolated and molecularly identified using *ITS* and *BenA* genes at the University of Belgrade. The species were maintained on Czapek agar slants and reinoculated every 7 days.

### Cultivation conditions and sample collection

The micromycetes were cultivated in two stages. To obtain pre-culture, fungal spores were washed off from a 7-day-old slant and transferred to a 750-ml shaker flask with 100 ml of media of the following composition, % (w/v): wort: 6.7, glucose: 1.0, peptone: 0.1, pH 5.5-6.0. The pre-cultures were placed in a shaker incubator at 28 °C (200 rpm). After 48 h, 3% (v/v) of the pre-culture was transferred to 100 ml of fermentation media №1 (% [w/v]: glycerol: 7.0, glucose: 3.0, fish flour hydrolysate: 3.0, NaNO_3_: 0.2, and MgSO_4_: 0.1) or fermentation media №2 (% (w/v): starch: 1.0, glucose: 3.5, fish flour hydrolysate: 0.5, peptone: 0.5, MgSO_4_: 0.05, KH_2_PO_4_: 0.05, NaCl: 0.2) and cultivated under the same conditions for 4 days (96 h). To obtain culture liquid, the contents of the flasks were filtered from the mycelium (using a funnel from Merck Millipore, USA) and stored at 4°C until further manipulations.

For every sample, the protein content was determined by Bradford assay [ 14 ] and several proteolytic activities, similar to those exposed by proteases of the human hemostasis system.

### Activity determination

Chromogenic peptide substrates were used to determine a spectrum of fungal proteolytic activities [ 15 ]. Thrombin- (Chromozym^TH^, S-2238), plasmin- (S-2251), factor Xa- (S-2765 and S-2222), urokinase- (S-2444), and protein C-like (S-2366) activities were assessed. To perform the reaction, 200 μl of the sample was added to 50 μl of 0.05 M Tris-HCl, pH 8.2, and 100 μl of the substrate (0.05%, prepared on the same buffer). The reaction was performed in a thermoshaker at 37°C with 600 rpm, and after 5 min of incubation, it was stopped with 200 μl of 50% acetic acid. The absorbance was measured at 405 nm. 

The activating activities towards proteins of the hemostatic system were also assessed for the samples that did not show any direct activity using a method established by V.G. Kreyer [ 16 ]. To determine the activating activity, 200 μl of the sample was preincubated with 50 μl of double-diluted human blood plasma in a thermoshaker at 37°C with 600 rpm. After 5 min of incubation, 100 μl of the 0.05% substrate in 0.05M Tris-HCl, pH 8.2 was added. After another 5 min of incubation under the same conditions, the reaction was stopped by adding 200 μl of 50% acetic acid. The absorbance was measured at 405 nm. 

One unit of activity was defined as the amount of p-nitroaniline (μmol) released in 1 ml of the sample for 1 min. In addition, fibrinolytic- and plasminogen-activating activities were assessed by Astrup–Müllertz–Hansen method with fibrin plates. Briefly, to prepare a fibrin plate, 9 ml of 0.47% bovine fibrinogen solution and 0.2 ml of 0.4% thrombin solution (both prepared on 0.9% NaCl) were mixed and plated in a glass Petri dish to polymerize. 

After 1 h of stabilization, one plate was heated for 30 min at 86 °C to inactivate the admixture of plasminogen. Samples (30 μL) were applied vertically on top of the plate to form drops, and the plates were incubated at 37°C for 3 h. After incubation, the diameters of hydrolysis zones were measured for both heated and unheated dishes. To calculate plasminogen-activating activity, the diameter of the dent on the heated dish was subtracted from the unheated one. A unit of activity was defined as a hydrolysis zone with a surface square of 1 mm^2^. 

## Results

For every sample, thrombin-, plasmin-, factor Xa-, urokinase-, and protein C-like activities using seven chromogenic peptide substrates were determined. For each substrate, species capable of effective proteolysis were found. The results are summarized in Table 1.

**Table 1:** Direct proteolytic activities of tested species towards chromogenic peptide substrates of the hemostasis system.

*Aspergillus* species	Fermentation medium	Activity, U×10^-3^						
		Chromozym^TH^	S-2238	S-2251	S-2765	S-2222	S-2444	S-2366
*A. aureolatus* BEOFB3320m	1	52.98±8.56	9.31±0.28	25.23±2.26	33.64±2.32	4.00±0.99	35.35±4.26	40.75±6.82
2	53.88±19.08	9.28±2.26	116.17±15.69	76.27±6.55	8.96±0.38	85.99±1.60	60.61±17.86
*A. calidoustus* BEOFB3220m	1	2.55±0.17	3.54±3.54	2.64±0.73	3.51±0.38	7.69±0.20	0.00	7.66±0.64
	2	6.84±1.04	1.97±0.64	0.00	1.16±1.16	1.80±1.68	1.48±0.38	9.74±0.99
*A. creber* BEOFB3250m	1	4.55±0.38	0.00	4.00±0.00	7.63±0.03	0.70±0.70	1.71±0.26	9.72±2.58
	2	20.97±0.73	8.73±0.26	6.12±0.38	15.69±1.60	3.68±0.38	6.26±0.93	62.90±2.12
*A. europaeus* BEOFB382m	1	0.00	3.02±0.22	0.35±0.05	0.32±0.32	1.57±0.70	0.75±0.23	0.00
	2	1.31±0.38	2.52±0.20	1.10±0.25	0.35±0.35	1.31±0.03	0.00	1.62±0.12
*A. jensenii* BEOFB3200m	1	15.57±0.38	7.16±1.17	5.05±0.29	13.80±5.45	1.12±0.24	1.51±0.12	47.56±3.94
	2	24.07±1.10	6.35±0.65	9.08±1.31	23.95±1.04	11.10±9.38	4.81±0.12	65.51±2.76
*A. melleus* BEOFB3180m	1	36.80±0.44	3.07±3.07	68.27±15.02	0.00	1.02±0.32	1.65±0.55	0.00
	2	55.85±5.68	5.63±2.44	69.98±6.58	3.05±0.84	16.97±0.15	5.19±1.02	1.07±1.07
*A. penicilloides* BEOFB3190m	1	2.49±0.52	0.70±0.08	0.84±0.04	3.34±0.03	2.84±0.17	0.00	0.00
	2	1.60±0.09	0.96±0.15	1.04±0.12	2.58±0.03	2.49±0.17	0.00	0.38±0.20
*A. proliferans* BEOFB3280m	1	1.54±0.20	1.13±0.13	0.00	4.15±0.90	0.70±0.00	0.64±0.04	4.29±0.06
	2	3.68±0.09	0.41±0.41	0.00	1.57±0.06	1.62±0.00	0.52±0.17	0.00
*A. protuberus* BEOFB3240m	1	0.00	15.08±15.08	6.93±0.26	19.98±5.02	6.90±0.52	0.00	65.22±0.38
	2	0.55±0.05	0.00	0.00	2.18±0.03	0.12±0.12	0.00	73.37±7.37
*A. pseudoglaucus* BEOFB3170m	1	0.44±0.12	0.29±0.06	0.46±0.12	2.00±0.38	0.00	5.51±0.06	2.20±0.81
	2	1.13±0.09	3.16±0.56	1.02±0.84	0.00	1.36±0.20	0.87±0.17	1.60±0.44
*A. domesticus* BEOFB3270m	1	1.51±0.75	1.39±0.09	0.00	2.49±0.45	2.64±1.02	0.81±0.01	1.02±0.04
	2	1.28±0.23	8.00±1.62	1.04±0.06	0.38±0.03	0.49±0.08	0.49±0.03	1.39±0.93
*A. ruber* BEOFB3150m	1	0.00	0.00	0.00	2.06±0.09	0.49±0.04	0.00	68.59±5.36
	2	1.42±0.38	0.00	0.00	1.33±0.81	2.35±0.38	0.00	111.85±2.52
*A. tabacinus* BEOFB3260m	1	50.37±10.59	17.05±17.05	9.40±0.93	31.93±7.16	6.99±1.25	15.43±3.89	69.46±5.65
	2	0.00	3.92±2.64	0.00	1.51±0.02	1.80±0.12	0.00	9.86±1.62
*A. tennesseensis* BEOFB3310m	1	53.33±1.19	8.90±8.90	25.52±9.05	34.19±0.38	4.15±0.09	10.03±0.87	52.69±7.11
	2	76.21±2.49	9.19±0.03	87.09±5.08	77.92±4.79	5.80±0.00	59.91±44.44	80.10±2.73
*A. tubingensis* BEOFB3300m	1	0.00	1.19±0.19	1.83±0.03	2.23±0.67	4.12±0.46	0.81±0.01	0.96±0.06
	2	4.47±0.46	12.59±10.6	3.02±0.87	1.10±0.35	1.57±0.00	1.57±0.23	2.52±0.67

No or relatively low direct activities were demonstrated for *A. calidoustus*, *A. europaeus*, *A. penicilloides*, *A. proliferans*, *A. pseudoglaucus*, *A. domesticus*, and *A. tubingensis*. High thrombin-like activity with Chromozym^TH^ was shown for *A. aureolatus* (52.98 U×10^-3^, medium №1; 53.88 U×10^-3^, and medium №2), *A. tabacinus* (50.37 U×10^-3^, medium №1), and *A. tennesseensis* (53.33 U×10^-3^, medium №1; 76.21 U×10^-3^, medium №2). The former and the latter species also showed the highest plasmin-, protein C-, and factor Xa-like as well as urokinase activities. 

Factor Xa-like activity of *A. tabacinus*, (31.93 U×10^-3^) was also significant. Besides the listed species, protein C-like activity was noticeable for *A. creber*, *A. jensenii*, *A. protuberus*, and *A. ruber*. These species showed high specificity towards the substrate of protein C. For species with low direct activities, activating activities were also determined, and the results are tabulated in Table 2. 

**Table 2:** Activating proteolytic activities of tested species towards chromogenic peptide substrates of the hemostasis system

*Aspergillus* species	Fermentation medium	Activity with the substrate. U×10^-3^						
		Chromozym ^TH^	S-2238	S-2251	S-2765	S-2222	S-2444	S-2366
*A. aureolatus* BEOFB3320m	1	-	-	0.00	1.54±0.00	1.16±0.09	-	-
	2	-	-	0.00	-	0.00	-	-
*A. calidoustus* BEOFB3220m	1	-	-	3.97±0.29	1.65±0.07	-	-	-
	2	-	0.00	0.00	0.00	0.00	0.00	-
*A. creber* BEOFB3250m	1	-	5.39±0.26	0.00	-	1.13±0.96	4.76±0.14	-
	2	-	-	0.00	-	0.44±0.35	0.00	-
*A. europaeus* BEOFB382m	1	2.76±0.26	-	1.16±0.06	11.51±0.12	0.00	6.79±0.96	5.51±0.12
	2	3.98±1.50	-	0.32±0.02	10.03±0.87	0.20±0.14	1.83±0.41	7.40±0.12
*A. jensenii* BEOFB3200m	1	-	-	-	-	0.00	0.87±0.08	-
	2	-	-	-	-	-	-	-
*A. melleus* BEOFB3180m	1	-	2.73±0.27	-	0.00	5.34±0.52	1.45±0.15	1.39±0.41
	2	-	-	-	0.00	2.23±0.83	41.59±0.92
*A. penicilloides* BEOFB3190m	1	-	0.00	0.00	-	-	0.00	0.00
	2	-	0.00	0.00	-	-	0.00	0.00
*A. proliferans* BEOFB3280m*	1	0.00	0.96±0.07	0.00	2.73±0.11	2.67±0.96	0.73±0.09	1.97±0.63
	2	5.57±0.58	4.12±0.11	0.00	2.70±0.38	4.52±0.25	3.97±0.84	0.52±0.03
*A. protuberus* BEOFB3240m	1	0.00	-	-	-	-	6.73±0.45	-
	2	1.42±0.20	1.94±0.10	2.00±0.00	3.83±0.26	0.00	3.92±0.23	-
*A. pseudoglaucus* BEOFB3170m	1	4.35±0.29	5.25±0.25	2.87±2.15	10.99±0.98	2.09±0.58	-	1.39±0.12
	2	2.84±0.06	2.81±0.30	2.29±1.94	3.10±0.36	2.41±0.10	-	3.68±0.73
*A. domesticus* BEOFB3270m	1	3.02±0.12	2.76±0.21	2.81±0.32	5.10±0.96	13.28±0.51	2.64±0.74	1.07±0.11
	2	1.31±0.21	-	11.02±0.06	2.03±0.24	9.28±0.97	1.71±0.14	2.38±0.24
*A. ruber* BEOFB3150m	1	-	0.00	0.00	5.02±0.95	0.00	3.02±0.06	-
	2	6.70±0.08	2.15±0.08	0.00	11.57±0.21	0.00	4.18±0.72	-
*A. tabacinus* BEOFB3260m	1	-	-	-	-	-	0.00	-
	2	0.00	-	0.00	-	4.00±0.14	1.62±0.61	-
*A. tennesseensis* BEOFB3310m	1	-	-	0.00	-	0.00	0.00	-
	2	-	-	0.00	-	0.00	0.00	0.00
*A. tubingensis* BEOFB3300m	1	1.42±0.60	2.84±0.14	1.19±0.08	3.92±0.48	9.19±0.47	4.32±0.36	2.09±0.11
	2	2.49±0.12	0.00	0.49±0.14	3.13±0.12	4.29±0.16	2.93±0.24	2.64±0.12

* The culture liquid pH appeared to be 2 and 3, hereby, the positive result was excluded, as in this case acid proteolysis is more likely to exist, compared to enzymatic proteolysis. In addition, the amount of detected protein in culture liquid was relatively low, which also proved the idea that the results are false-positive and no proteases are involved in fibrinolysis.

In general, all the species showed weak activating activities (Table 3). Only three fungi were capable of slight activating of human blood proteins. These were *A. ruber*, *A. domesticus*, and *A. melleus*. The latter is known as a protease producer since the end of the last century, and its protease is already well-studied. In this study, it only activated protein C (41.59 U×10^-3^, medium №2). *Aspergillus domesticus* activated plasmin (11.02 U×10^-3^, medium №2) and factor Xa (13.28 U×10^-3^, medium №1), while *A. ruber* appeared to have a slight (11.57 U×10^-3^, medium №2) activating activity only against factor Xa. No activating activities toward thrombin or urokinase were found.

**Table 3:** Fibrinolytic and plasminogen-activating activities of studied species assessed with fibrin plates.

*Aspergillus* species	Fibrinolytic activity, U		Plasminogen-activating activity, U	
	Fermentation medium 1	Fermentation medium 2	Fermentation medium 1	Fermentation medium 2
*A. aureolatus* BEOFB3320m	519.48±30.61	385.03±25.31	0.00	162.01±2.42
*A. calidoustus* BEOFB3220m	0.00	0.00	0.00	68.27±21.19
*A. creber* BEOFB3250m	0.00	198.14±16.48	0.00	163.17±32.96
*A. europaeus* BEOFB382m	0.00	91.58±11.77	48.29±2.35	164.83±49.45
*A. jensenii* BEOFB3200m	0.00	0.00	0.00	0.00
*A. melleus* BEOFB3180m	273.06±84.77	510.32±43.56	0.00	176.49±42.38
*A. penicilloides* BEOFB3190m	0.00	0.00	83.25±11.77	86.58±47.09
*A. proliferans* BEOFB3280m*	0.00*	0.00*	0.00*	0.00*
*A. protuberus* BEOFB3240m	0.00	188.15±35.32	29.97±7.06	241.43±40.03
*A. pseudoglaucus* BEOFB3170m	0.00	0.00	0.00	0.00
*A. domesticus* BEOFB3270m	0.00	0.00	0.00	0.00
*A. ruber* BEOFB3150m	0.00	0.00	53.28±0.00	87.41±9.42
*A. tabacinus* BEOFB3260m	168.17±7.06	0.00	49.95±0.00	0.00
*A. tennessensis* BEOFB3310m	135.70±22.37	316.77±22.96	0.00	151.52±2.35
*A. tubingensis* BEOFB3300m	477.52±2.83	241.43±40.03	245.59±34.14	0.00

* The result is excluded as a false-positive, as described in Table 2.

Three species (*A. jensenii*, *A. pseudoglaucus*, and *A. domesticus*) did not show any fibrinolytic or plasminogen-activating activity. *Aspergillus proliferans* presumably produced organic acids, which were detected by measuring the pH of the culture liquid. 

Moreover, the blurred zones around the holes on fibrin plates, typical for acid hydrolysis, were registered. In combination with low determined protein content, we suggest the exclusion of these results as false-positive. 

Eight species have shown direct fibrinolytic activity. The highest activities were registered with samples obtained from *A. aureolatus* (519.48 U, medium №1), *A. melleus* (510.32 U, medium №2), and *A. tubingensis* (477.52 U, medium №1). *Aspergillus creber* (198.14 U), *A. protuberus* (188.15 U), and *A. europaeus* (91.58 U) only secreted fibrinolytics when cultivated on one media, with an amine nitrogen source. Otherwise, no activity was observed. In contrast, for *A. tabacinus* (168.17 U), a presence of mixed nitrogen in the nutrition media was found to be necessary to expose fibrinolysis. 

The majority of the species showed plasminogen-activating activity. The highest values were observed for *A. tubingensis* (245.59 U) and *A. protuberus* (241.43 U) cultivated on media 1 and 2, respectively. Sufficient but not high activities were shown for *A. europaeus*, *A. penicilloides*, and *A. ruber*.

## Discussion

In this study, the abilities of 15 novel *Aspergillus* species to produce proteases were screened, including activity against components of human hemostasis. The assessed activities included direct thrombin-, plasmin-, factor Xa-, urokinase-, and protein C-like-activities, as well as activating activities towards the aforementioned proteins with human blood plasma. This was determined using chromogenic peptide substrates in culture fluid samples obtained after submerged fermentation of native strains. Fibrinolytic and plasminogen-activating activities on fibrin plates were also determined.

*A. aureolatus* and *A. tennesseensis* showed high proteolytic activities against substrates of thrombin, plasmin, urokinase, and factor Xa. It is noteworthy that for *A. aureolatus*, plasmin-like activity (116.17 U×10^-3^, medium №2) was almost three times higher than for most promising species (*A. versicolor* 1, *A. terreus* 2) from previous works [ 8 , 17 ]. The urokinase activity of this strain also exceeded every value determined in previous studies, which illustrates the biotechnological potential of this species. It corresponded to 85.99 U×10^-3^ (medium 2) while for Sarocladium strictum 203, a described producer of proteases with urokinase activity, it was 58.3 U×10^-3^ [ 17 ]. Besides, for *A. tennesseensis*, the determined factor Xa-like activity (77.92 U×10^-3^) appeared to be higher than that of the previously described species.

Among the determined activating activities, only *A. melleus* should be highlighted. It is known that *A. melleus* protease is capable of activating Hageman factor, and in this study, the activating activity for protein C was shown for the first time. One of the proteases from *A. melleus* is commercially available, and activating activity for protein C may suggest that under described cultivation conditions this strain produces another protease.

Eight out of 15 (53.33%) species showed direct fibrinolytic activity. Four of them (*A. aureolatus*, *A. melleus*, *A. tennesseensis*, and *A. tubingensis*) were capable of producing fibrinolytic enzymes when cultivated with an amine nitrogen source, as well as with a mixed one (amine and nitrate). Other species only expressed desired activity when cultivated with either amine nitrogen (*A. creber*, *A. protuberus*, and *A. europaeus*) source or mixed (*A. tabacinus*). Furthermore, catabolic repression in protease production was observed for *A. creber*, *A. jensenii*, *A. penicilloides*, *A. ruber*, and *A. tennesseensis*. This data may be used for biotechnological production of other substances, synthesized by these strains, as well as for proper metabolism investigation.

A large number of the species (73.33%) showed plasminogen-activating activity. The *A. protuberus* as well as *A. europaeus*, *A. penicilloides*, and *A. ruber*, could secrete plasminogen activators when cultivated with both amine and mixed nitrogen sources. Among the studied, *A. aureolatus*, *A. creber*, *A. melleus*, *A. protuberus*, *A. tabacinus*, *A. tennesseensis*, and *A. tubingensis* exposed both direct and non-direct fibrinolysis on the same media, which may indicate a presence of non-specific protease in their culture liquid. However, for *A. calidoustus* (68.27 U), *A. penicilloides* (83.25 and 86.58 U on 1-st and 2-nd fermentation media, respectively), and *A. ruber* (53.28 and 87.41 U, same), only plasminogen-activating activity was registered, which makes them promising producers for pharmacy and biomedicine. 

For *A. proliferans*, incapable of effective enzymatic proteolysis, organic acid production and culture liquid acidification (to pH 2-3) were registered. Despite the existence of more registered positive activities for samples obtained after cultivation on media2, in some cases, they were not higher than those obtained on media1. This proves a diversity of physiological responses of *Aspergillus* fungi on varying nitrogen sources. Therefore, for every potential producer, it is necessary to optimize the composition of the nutrition medium in the future.

*Aspergillus aureolatus* and *A. tennesseensis* have shown high activity towards a significant number of substrates, and, hereby, low substrate specificity, which doubts their potential application in biomedicine and pharmaceutics. In contrast, *A. creber*, *A. jensenii*, and *A. protuberus* have shown outstanding protein-C-like activities, which proves them as possible agents for biomedicine and pharmacology. 

## Conclusion

Microbial proteases are promising for many fields of industry; however, their high specificity is key to the treatment of cardiovascular diseases. Producers of active enzymes are in high demand and are sought by many research groups in the world. One of the biotechnologically proven groups of producers is *Aspergillus* fungi. In this research, proteolytic activities against proteins of the hemostasis system were shown for 15 species of *Aspergillus* for the first time. *Aspergillus aureolatus* and *A. tennesseensis* have shown potential as producers of non-specific proteases, when *A. creber*, *A. jensenii*, and *A. protuberus* may be applied as producers of hemostatically active proteases for pharmacology and biomedicine.

## Acknowledgments

The authors would like to express their gratitude and appreciation to Biljana Nikolić and Željko Savković from the University of Belgrade for the provided strains. The authors also acknowledge the Grants Council of the President of the Russian Federation for providing funding for the work.

## Authors’ contribution

A.O. and A.S. conceptualized and supervised the study. V.L., D.S., and S.K. performed the experiments. A.S., D.S., and V.L. performed Data processing. A.S., D.S., and V.L. prepared the original draft. All authors have read and approved the final manuscript.

## Conflicts of interest

There was no conflict of interest between the authors of this study.

## Financial disclosure

This research was supported by the Grants Council of the President of the Russian Federation №СП-3906.2021.4.
